# Allspice and Clove As Source of Triterpene Acids Activating the G Protein-Coupled Bile Acid Receptor TGR5

**DOI:** 10.3389/fphar.2017.00468

**Published:** 2017-07-17

**Authors:** Angela Ladurner, Martin Zehl, Ulrike Grienke, Christoph Hofstadler, Nadina Faur, Fátima C. Pereira, David Berry, Verena M. Dirsch, Judith M. Rollinger

**Affiliations:** ^1^Department of Pharmacognosy, Faculty of Life Sciences, University of Vienna Vienna, Austria; ^2^Department of Pharmaceutical Chemistry, University of Vienna Vienna, Austria; ^3^Department of Analytical Chemistry, University of Vienna Vienna, Austria; ^4^Department of Microbiology and Ecosystem Science, University of Vienna Vienna, Austria

**Keywords:** TGR5, *Syzygium aromaticum*, *Pimenta dioica*, *Kaempferia galanga*, triterpene acids

## Abstract

Worldwide, metabolic diseases such as obesity and type 2 diabetes have reached epidemic proportions. A major regulator of metabolic processes that gained interest in recent years is the bile acid receptor TGR5 (Takeda G protein-coupled receptor 5). This G protein-coupled membrane receptor can be found predominantly in the intestine, where it is mainly responsible for the secretion of the incretins glucagon-like peptide 1 (GLP-1) and peptide YY (PYY). The aim of this study was (i) to identify plant extracts with TGR5-activating potential, (ii) to narrow down their activity to the responsible constituents, and (iii) to assess whether the intestinal microbiota produces transformed metabolites with a different activity profile. Chenodeoxycholic acid (CDCA) served as positive control for both, the applied cell-based luciferase reporter gene assay for TGR5 activity and the biotransformation assay using mouse fecal slurry. The suitability of the workflow was demonstrated by the biotransformation of CDCA to lithocholic acid resulting in a distinct increase in TGR5 activity. Based on a traditional Tibetan formula, 19 plant extracts were selected and investigated for TGR5 activation. Extracts from the commonly used spices *Syzygium aromaticum* (SaroE, clove), *Pimenta dioica* (PdioE, allspice), and *Kaempferia galanga* (KgalE, aromatic ginger) significantly increased TGR5 activity. After biotransformation, only KgalE showed significant differences in its metabolite profile, which, however, did not alter its TGR5 activity compared to non-transformed KgalE. UHPLC-HRMS (high-resolution mass spectrometry) analysis revealed triterpene acids (TTAs) as the main constituents of the extracts SaroE and PdioE. Identification and quantification of TTAs in these two extracts as well as comparison of their TGR5 activity with reconstituted TTA mixtures allowed the attribution of the TGR5 activity to TTAs. EC_50_s were determined for the main TTAs, i.e., oleanolic acid (2.2 ± 1.6 μM), ursolic acid (1.1 ± 0.2 μM), as well as for the hitherto unknown TGR5 activators corosolic acid (0.5 ± 1.0 μM) and maslinic acid (3.7 ± 0.7 μM). In conclusion, extracts of clove, allspice, and aromatic ginger activate TGR5, which might play a pivotal role in their therapeutic use for the treatment of metabolic diseases. Moreover, the TGR5 activation of SaroE and PdioE could be pinpointed solely to TTAs.

## Introduction

The bile acid receptor TGR5 [Takeda G protein-coupled receptor 5, also known as G protein-coupled bile acid receptor 1 (GPBAR1) or membrane-type receptor for bile acids (M-BAR)] is expressed in a wide variety of tissues and cell types including the intestine, gallbladder, adipocytes, and immune cells and is considered to be a major regulator in metabolism. TGR5 acts in a tissue-specific manner *via* increasing intracellular levels of cAMP and thereby modulating several downstream signaling pathways (Kawamata et al., 2003; Thomas et al., 2008). Activation of TGR5 has been shown to enhance insulin secretion in the pancreas (Kumar et al., 2012) as well as gallbladder filling (Li et al., 2011). In addition, TGR5 influences bile acid pool size and composition (Maruyama et al., 2006) and has been shown to be nephroprotective in models of diabetes and obesity (Wang et al., 2016). In immune cells, TGR5 agonists show anti-inflammatory activities (Wang et al., 2011; Yoneno et al., 2013), whereas in enteroendocrine cells, release of the incretins glucagon-like peptide 1 (GLP-1) and peptide YY (PYY) is stimulated (Thomas et al., 2009; Harach et al., 2012; Bala et al., 2014). Furthermore, in muscle and brown adipose tissue, TGR5 agonists are able to increase energy expenditure (Watanabe et al., 2006; Svensson et al., 2013; Broeders et al., 2015) and in the enteric nervous system, gastric emptying is delayed and gut motility decreased (Poole et al., 2010). This multitude of effects renders TGR5 a promising target for the treatment of metabolic diseases (Perino and Schoonjans, 2015).

This prompted us to select and study plant extracts derived from a Tibetan herbal preparation, which is used against different symptoms of metabolic diseases (Brunner-La Rocca et al., 2005; Melzer et al., 2006; Melzer and Saller, 2013; Schwabl and Vennos, 2015).

Since TGR5 is abundantly expressed in the intestine, which facilitates local interactions between plant constituents and various components present in the gut, we also considered the possibility of microbiota-mediated biotransformation of extract constituents. Biotransformation by microorganisms residing in the gastrointestinal tract holds significant potential for the discovery of bioactive compounds (Saha et al., 2016). Components of food or herbal drugs that are not absorbed in the upper intestinal tract are able to reach the large intestine, where most of the gut microbiome resides (Aron-Wisnewsky et al., 2012). Gut microorganisms possess an arsenal of enzymes that are able to chemically modify a large variety of compounds, including plant secondary metabolites, thereby allowing the formation of new compounds with potentially diverging bioactivities. Typical examples for such enzyme-catalyzed reactions are the hydrolysis of glycosides (e.g., by α-rhamnosidase, β-glucuronidase, β-glucosidase) and esters (e.g., by sulfatases, carboxylesterase), reduction, oxidation, dehydroxylation, demethylation, decarboxylation, dehydrogenation, and various isomerizations (Possemiers et al., 2011).

Thus, the aims of this study were (i) to identify plant extracts which are able to activate TGR5 *in vitro*, (ii) to identify the active components in the respective original and biotransformed plant extracts, and (iii) to monitor the metabolite profile of TGR5-activating extracts considering putative biotransformation of the herbal constituents by incubation with mouse fecal microbiota.

## Materials and Methods

### Reference and Standard Compounds

Maslinic acid, corosolic acid, oleanolic acid, and ursolic acid were obtained from Phytolab (Vestenbergsgreuth, Germany), lithocholic acid (LCA), chenodeoxycholic acid (CDCA), oleic acid, and palmitic acid from Sigma Aldrich (St. Louis, MO, United States), stearic acid and 4-methoxycinnamic acid from Fluka (Buchs, Switzerland), chlorogenic acid from Carl Roth (Karlsruhe, Germany), and linoleic acid from Acros (Geel, Belgium). Eugenol was isolated from clove and the identity and purity tested by ATR-IR spectroscopy. All reference and standard compounds were obtained in a purity of ≥90%. They were dissolved to 1.0 mg/mL in MeOH. The triterpene acids (TTAs) used for the quantification were mixed and diluted to give a standard solution containing 100 μg/mL of each corosolic acid, maslinic acid, oleanolic acid, and ursolic acid followed by a serial dilution down to 2 μg/mL.

### Cell Culture Reagents, Chemicals, and Plasmids

Dulbecco’s modified Eagle’s medium (DMEM), containing 4.5 g/L glucose, L-glutamine, benzylpenicillin, and streptomycin were obtained from Lonza (Basel, Switzerland). Fetal bovine serum (FBS) and trypsin was supplied by Gibco *via* Invitrogen (Lofer, Austria). The TGR5 expression plasmid, GPBAR1, transcript variant 3, NM_170699.1 (OG-SC123315) was obtained from Origene via Biomedica (Vienna, Austria). The CRE-Luc plasmid, pGL4.29[luc2P/CRE/Hygro], was obtained from Promega (Mannheim, Germany) and the plasmid encoding enhanced green fluorescence protein (pEGFP-N1) was obtained from Clontech (Mountain View, CA, United States).

### Plant Material

Dried and powdered herbal material was kindly provided by PADMA AG (Wetzikon, Switzerland). Information regarding the used organ, plant species, batch number, and acronym of the prepared extracts are given in **Table 1**. Voucher specimens are deposited at the Department of Pharmacognosy, University of Vienna, Austria.

**Table 1:** Information regarding the used plant material for extract preparation (including extract acronym).

Acronym of the extract	Plant species	Used organ	English name	Batch number
AmarE	*Aegle marmelos* (L.) Correa	Half-ripe entire fruits	Bael tree fruit	20982301
AvulE	*Aquilegia vulgaris* L.	Aerial parts	Columbine	21290300
AindE	*Azadirachta indica* A. Juss	Endocarp and seeds	Neem fruit	21108301
CoffE	*Calendula officinalis* L.	Entire flowering part	Marigold	21348300
CislE	*Cetraria islandica* (L.) Acharius s.l.	Thallus	Iceland moss	20885300
EcarE	*Elettaria cardamomum* (Roxb.) Maton var. *minuscula* Burkill	Entire fruits (including seeds)	Cardamom fruit	21391101
GglaE	*Glycyrrhiza glabra* L.	Roots and stolons	Liquorice root	21392100
KgalE	*Kaempferia galanga* L.	Rhizomes	aromatic ginger	21345300
LsatE	*Lactuca sativa* var. *capitata* L.	Leaves	Lettuce leave	21400300
PlanE	*Plantago lanceolata* L. s.l.	Leaf and scape	Ribwort plantain	21327101
PdioE	*Pimenta dioica* (L.) Merr.	Unripe fruits	Allspice	21362100
PaviE	*Polygonum aviculare* L. s.l.	Aerial parts	Knotgrass	21322100
PaurE	*Potentilla aurea* L.	Aerial parts	Potentilla golden herb	21161301
PsanE	*Pterocarpus santalinus* L.f.	Heartwood	Red sandalwood	20712307
ScosE	*Saussurea costus* (Falc.) Lipschitz	Roots	Costus root	21280300
ScorE	*Sida cordifolia* L.	Aerial parts	Heart-leaved sida	20981300
SaroE	*Syzygium aromaticum* (L.) Merr. & L. M. Perry	Flower buds	Clove	21321101
TcheE	*Terminalia chebula* Rertz	Fruits	Myrobalan fruit	21324301
VoffE	*Valeriana officinalis* L. s.l.	Rhizomes, roots and stolons	Valerian root	21388100

### Extraction and Tannin Depletion

Small scale extraction was adapted from Camp et al. (2012) as previously described (Kratz et al., 2016). In brief, 300 mg plant material was defatted with *n*-hexane before subsequent extraction with CH_2_Cl_2_ (7 mL) and MeOH (13 mL) for 15 min each by ultrasonication. The combined and dried extracts were resuspended in 4 mL MeOH and loaded onto a SPE polyamide gel cartridge for tannin depletion. The flow-through and eluate from washing with 2 × 4 mL MeOH was combined and dried under reduced pressure. Dried extracts were dissolved in dimethyl sulfoxide (DMSO) prior to bioactivity measurements. For bioassays, the final DMSO concentration was 0.1%.

### Fractionation of the *Kaempferia galanga* Extract (KgalE)

To produce sufficient amounts of extract for further fractionation, the whole extraction and tannin-depletion procedure (see above) was scaled up by a factor of 10. The obtained extract (KgalE, 70 mg) was then fractionated by flash chromatography (PuriFlash 4250 system, Interchim, Montlucon Cedex, France) on a PuriFlash column (PF-RPC18HQ, 15 μm, 6g, Interchim) using water and acetonitrile as mobile phases A and B, respectively. The sample was applied as dry load onto the PuriFlash column and eluted at 25°C with a flow rate of 5 mL/min and a linear gradient of 5–95% B in 45 min. Eluting compounds were detected with both, a UV-detector (200–400 nm) and an evaporative light-scattering detector (ELSD). Six fractions (KgalE-1 to 6) were collected based on the ELSD signal followed by TLC monitoring (stationary phase: Merck silica gel 60 PF_254_; mobile phase: toluol/ethyl acetate/formic acid/methanol/acetone, 4:2:1:0.5:0.25:0.25; detection with staining reagents vanillin/H_2_SO_4_ at vis, UV_254_, UV_366_), with the following elution volumes: KgalE-1, 90–138 mL; KgalE-2, 138–204 mL; KgalE-3, 204–237 mL; KgalE-4, 237–261 mL; KgalE-5, 267–327 mL; KgalE-6, 327–351 mL.

### Preparation and Analysis of the Essential oil of *K. galanga*

Essential oil of *K. galanga* was obtained by small-scale steam distillation of 2.0 g of the dried and pulverized drug with 20 mL of water for 1.5 h. The main constituents of the volatile oil were identified by GC-MS (mass spectrometry) on a QP2010 System (Shimadzu Austria, Korneuburg, Austria) equipped with a Zebron ZB-5 capillary column (60 m length, 0.25 μm film thickness; Phenomenex, Aschaffenburg, Germany). One microliter of the diluted essential oil (1:100 in CH_2_Cl_2_) was injected and separated with He (5.0) as carrier gas applying a temperature gradient from 50 to 270°C in 45 min. EI mass spectra were recorded in the range of *m/z* 40–500. The chemical composition in terms of normalized % peak area was determined on an AutoSystem XL equipped with a SE-54-CB capillary column (10 m length, 0.25 μm film thickness) and an FID detector. In this case, N_2_ (5.0) was used as carrier gas and the temperature was increased from 50 to 155°C in 35 min.

### Biotransformation of Plant Extracts by Mouse Fecal Slurry

Simulated gastric fluid (SGF) was prepared according to the European Pharmacopoeia (Ph. Eur. 8th): 2.0 g NaCl were dissolved in water. Eighty milliliters of 1 M HCl were added and the solution was diluted with water to achieve 1000 mL solution (pH = 1.2). Neutralization to a pH of ∼7 after incubation was achieved by adding diluted NaOH (Ph. Eur. 8; 8.5 g NaOH dissolved in water to achieve a 100 mL solution). Phosphate-buffered saline (PBS) was prepared with 137 mM NaCl, 2.7 mM KCl, 10 mM Na_2_HPO_4_, and 1.8 mM KH_2_PO_4_ to achieve a pH of 7.4.

Fecal slurry was prepared with fecal pellets obtained from C57BL/6 wild type mice (kindly provided by the Max F. Perutz Laboratories Animal Facility, Vienna). All procedures involving animal handling and sampling were performed according to protocols approved by the Austrian law (BMWF 68.205/0032-WF/II/3b/2014). Handling of the animals for sample collection was carried out by FELASA B degree holding personnel. Freshly evacuated fecal pellets were collected from mice that were housed together in the same cage, and immediately stored into an Oxoid^TM^ Anaerobic 3.5 L jar, where oxygen is removed by the presence of a AnaeroGen sachet (Thermo Scientific AN0035). The jar containing the fecal pellets was then introduced in a Vinyl Anaerobic Chamber (Coy Laboratory Products). Fecal pellets (∼12) were resuspended in 18 mL of PBS, and used instantly after preparation. For control samples (labeled with superscript 0), suspended fecal slurry was autoclaved before incubation.

Dried tannin-depleted extracts (4 mg) or CDCA (100 nmol) were incubated with 1 mL SGF for 90 min at 37°C followed by neutralization with diluted NaOH. Then, 1 mL of either autoclaved or non-autoclaved fecal slurry was added followed by incubation for 24 h at 37°C under anaerobic conditions. The incubation was terminated by adding ethanol to a final concentration of 48 vol% ethanol followed by storage at -20°C for about 18 h. Centrifugation and sterile filtration were applied before subjecting the samples to analytical and pharmacological investigations.

### Identification of the Main Constituents in the Extracts and Fractions by HPLC-DAD-CAD and LC-MS

To identify the TGR5-activating compounds, samples were analyzed by high-performance liquid chromatography (HPLC) with charged aerosol detection (CAD) and HPLC-MS. These analyses were performed on an UltiMate 3000 RSLC-series system (Dionex/Thermo Fisher Scientific, Germering, Germany) coupled in parallel to a Corona ultra RS charged aerosol detector (CAD, Dionex/Thermo Fisher Scientific) and an HCT 3D quadrupole ion trap mass spectrometer equipped with an orthogonal ESI source (Bruker Daltonics, Bremen, Germany). Separation was carried out on an Acclaim 120 C18, 2.1 mm × 150 mm, 3 μm HPLC column (Dionex/Thermo Fisher Scientific) using 0.1% aqueous formic acid and acetonitrile as mobile phase A and B, respectively. The gradient was slightly modified for each extract (*Syzygium aromaticum*: 5–25% B in 10 min, 25–65% B in 8 min, 65–89% B in 12 min, and 89–95% B in 1 min; *Pimenta dioica*: 5–26% B in 14 min, 26–75% B in 14 min, and 75–95% B in 10 min; *K. galanga*: 5–95% B in 45 min). Each gradient was followed by a washing (10 min at 95% B) and re-equilibration step (10 min at 5% B). The flow rate was 0.5 mL/min and the column oven temperature was set to 25°C. After passing the diode-array detector (DAD), the eluate flow was split 4:1 between the CAD and the MS, respectively. The CAD nebulizer temperature was 35°C and the ESI ion source was operated as follows: capillary voltage: +3.5/-3.7 kV, nebulizer: 26 psi (N_2_), dry gas flow: 9 L/min (N_2_), and dry temperature: 340°C. Positive and negative ion mode multistage mass spectra up to MS^3^ were obtained in automated data-dependent acquisition (DDA) mode using helium as collision gas, an isolation window of Δ*m/z* = 4, and a fragmentation amplitude of 1.0 V.

To confirm the tentative identifications achieved with the above system, high-resolution mass spectra were recorded on a maXis HD ESI-Qq-TOF mass spectrometer (Bruker Daltonics) that was also connected to an UltiMate 3000 RSLC-series system. The separation was performed with the above-described HPLC methods. The eluate flow was split approximately 1:8 and the following ESI ion source settings were applied: capillary voltage: ±4.5 kV, nebulizer: 0.8 bar (N_2_), dry gas flow: 7.0 L/min (N_2_), and dry temperature: 200°C. The sum formulas of the detected ions were determined using Bruker Compass DataAnalysis 4.2 based on the mass accuracy (Δ*m/z* ≤ 10 ppm) and isotopic pattern matching (SmartFormula algorithm).

### Quantification of the Main Triterpene Acids in Extracts of *S. aromaticum* (SaroE) and *P. dioica* (PdioE)

The main TTAs in SaroE and PdioE were quantified with the above-described HPLC-DAD-CAD system using external calibration. While the same stationary and mobile phase was applied as for the identification, other parameters had to be adapted to achieve better separation of the analytes: the flow rate was set to 0.3 mL/min, the column oven temperature to 10°C; the gradient consisted of a linear increase from 80 to 91% B in 22 min (followed by a column cleaning and a re-equilibration step). The extracts, quantification standards (100, 40, 20, 10, 5, and 2 μg/mL each of corosolic acid, maslinic acid, oleanolic acid, and ursolic acid), a quality control sample (12 μg/mL each of corosolic acid, maslinic acid, oleanolic acid, and ursolic acid) and a blank sample (pure MeOH) were analyzed in quadruplicates. For each analyte, the peak area was obtained from the CAD chromatograms and a linear calibration function was determined from the quantification standards, which was then used to calculate the concentration of the four TTAs in the active extracts.

### TGR5 Luciferase Reporter Gene Assay

For the TGR5 luciferase reporter gene assay HEK-293 cells (ATCC, Manassas, VA, United States) were maintained in DMEM containing 4.5 g/L glucose supplemented with 2 mM glutamine, 10% FBS, 100 U/mL benzylpenicillin and 100 μg/mL streptomycin. Cells were grown in 15 cm dishes at a density of 6 × 10^6^ cells per dish for 19 h and then transfected *via* the calcium phosphate precipitation method with 5 μg TGR5 expression plasmid, 5 μg CRE-Luc plasmid, and 3 μg of a plasmid encoding EGFP as internal control. After 6 h the cells were reseeded to 96-well plates (5 × 10^4^ cells/well) in DMEM containing 4.5 g/L glucose supplemented with 2 mM glutamine, 100 U/mL benzylpenicillin, 100 μg/mL streptomycin, and 5% charcoal-stripped FBS. Cells were treated with indicated concentrations of test extracts, positive control (10 μM LCA) or solvent vehicle (0.1% DMSO) and incubated for 18 h. Cells were then lysed and the luminescence of the firefly luciferase and the fluorescence of EGFP were quantified with a GeniosPro plate reader (Tecan, Grödig, Austria). The luciferase-derived luminescence was normalized to the EGFP-derived fluorescence from each well to account for differences in transfection efficiency or cell number.

### Statistical Analysis

Statistical analyses were performed with the GraphPad prism software version 4.03. Non-linear regression (sigmoidal dose response) was used to calculate the EC_50_ values and maximal fold activation. The data are presented as the mean ± SD. Statistical evaluation was performed by one-way analysis of variance (ANOVA) and Student’s *t*-test. Differences between groups with a *p*-value <0.05 were considered statistically significant.

## Results

### Plant Selection

Nineteen herbal drugs of a Tibetan formula used against different symptoms associated with the metabolic syndrome were selected and tested for their potential to activate TGR5. The formula contains the pulverized material of the following plant materials: costus root, Iceland moss, neem fruit, cardamom fruit, myrobalan fruit, red sandalwood, allspice, bael tree fruit, columbine, liquorice root, ribwort plantain, knotgrass, potentilla golden herb, clove, aromatic ginger, heart-leaved sida, valerian root, lettuce leaf, and marigold.

In accordance to the oral application of the pulverized material, extracts were prepared as previously described (Kratz et al., 2016) by a protocol adapted from Camp et al. (2012). Herein, defatted plant material is extracted successively with dichloromethane and methanol. The two resulting extracts were combined in order to cover drug-like properties in a wide range of polarity. Further, tannin depletion via polyamide gel was carried out in order to remove common assay-interfering compounds.

### TGR5 Activation by Extracts of *P. dioica* (PdioE), *S. aromaticum* (SaroE), and *K. galanga* (KgalE)

The obtained extracts were screened in HEK-293 cells transfected with the TGR5 receptor and a luciferase gene containing the respective response element in its promoter (CRE-Luc). Three plant extracts were able to significantly activate TGR5 at concentrations of 100 and 30 μg/mL, namely those of the dried flower buds of *S. aromaticum* (SaroE), commonly known as cloves, the unripe fruits of *P. dioica* (PdioE), commonly known as allspice, and the rhizomes of *K. galanga* (KgalE), known as aromatic ginger. These three medicinal plants are used as herbal remedies for the treatment of digestive problems, diabetes, as antioxidants and analgesics in traditional medicines, especially of Middle Eastern and Asian countries as well as the Caribbean (Achuthan and Padikkala, 1997; Zhang and Lokeshwar, 2012; Kumar et al., 2013; Cortes-Rojas et al., 2014; Opara and Chohan, 2014; Sanae et al., 2014; Tu et al., 2014). Interestingly, all three herbal drugs are also widely used as spices.

**FIGURE 1:**
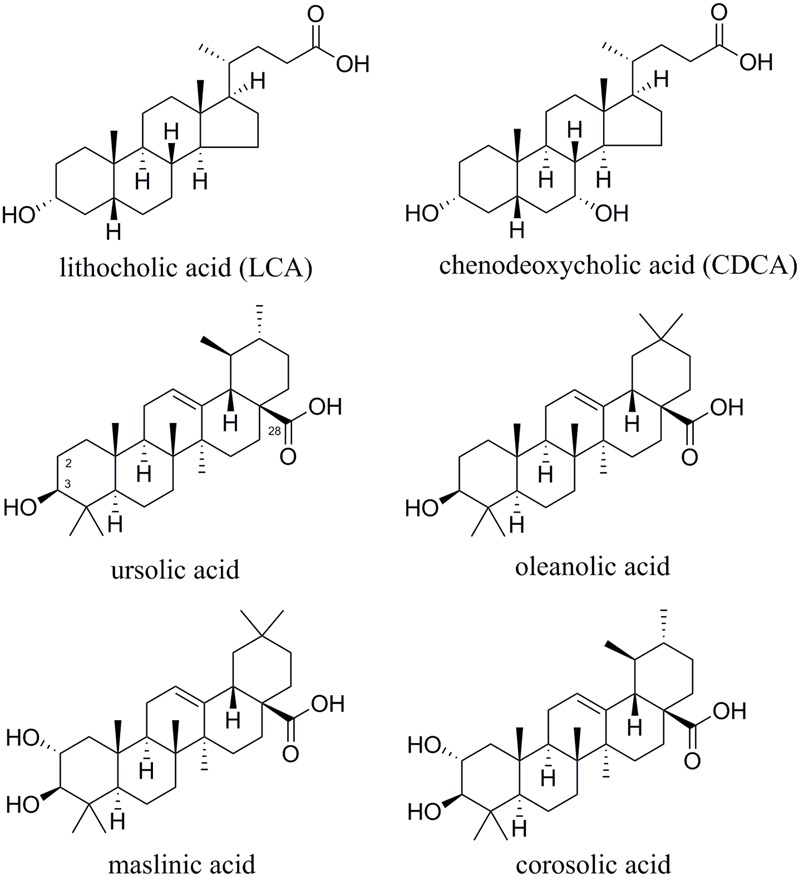
Chemical structures of lithocholic acid (LCA), chenodeoxycholic acid (CDCA), and TTAs.

**FIGURE 2:**
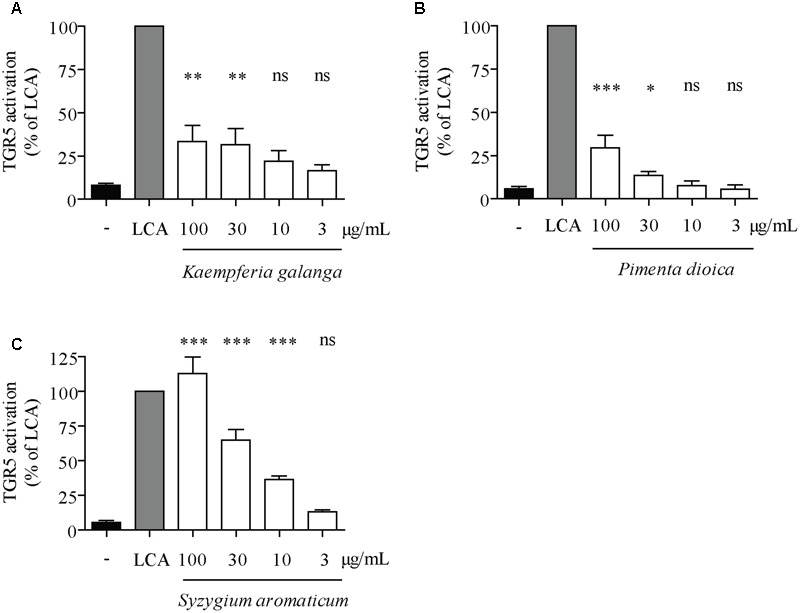
Concentration–response studies with plant extracts regarding TGR5 activity. HEK-293 cells were cotransfected with a TGR5 expression plasmid, a CRE luciferase reporter plasmid and an EGFP plasmid as internal control. Cells were treated with 10 μM lithocholic acid (LCA) as positive control or the indicated concentrations of **(A)** PdioE, **(B)** SaroE, or **(C)** KgalE for 18 h. The measured luciferase-derived luminescence was normalized to the obtained EGFP-derived fluorescence. Results are expressed as % activation compared to the reference LCA. Each bar represents the mean ± SD of at least three independent experiments performed in quadruplicate and evaluated by one-way ANOVA with the Bonferroni post-test. ^∗∗∗^*p* < 0.001, ^∗∗^*p* < 0.01, ^∗^*p* < 0.05 compared with solvent vehicle control (DMSO), ns not significant versus DMSO.

Concentration–response studies were performed with the three TGR5-activating extracts PdioE, SaroE, and KgalE to determine their EC_50_ and *E*_max_ values. As reference compound, the well-established TGR5 agonist LCA (**Figure 1**) was used at a concentration of 10 μM, which was set as 100% TGR5 activation. The assay was validated with CDCA (**Figure 1**), which showed an EC_50_ of 33.2 ± 4.5 μM with 34.6 ± 8.6% maximum relative TGR5 activation in our assay. All three plant extracts were able to increase TGR5 activity in a concentration-dependent manner; KgalE showed the lowest EC_50_ with 12.9 ± 24.1 μg/mL and an *E*_max_ of 33.2 ± 4.5% compared to LCA (**Figure 2A** and **Table 2**). PdioE elicited an *E*_max_ of 29.5 ± 8.3% compared to LCA and the highest EC_50_ of all three plant extracts with 60.2 ± 13.7 μg/mL (**Figure 2B**). Intriguingly, SaroE revealed the highest efficiency with an *E*_max_ of 112.8 ± 12.1% compared to LCA, overreaching by far the efficiency of our positive control CDCA. The EC_50_ of SaroE was determined to be 19.8 ± 16.6 μg/mL (**Figure 2C** and **Table 2**).

**Table 2:** EC_50_ and *E*_max_ values of extracts from *P. dioica* (PdioE), *S. aromaticum* (SaroE), and *K. galanga* (KgalE) compared to chenodeoxycholic acid (CDCA).

	EC_50_ (μg/mL) or (μM)^∗^	*E*_max_ (% LCA)
PdioE	60.2 ± 13.7	29.5 ± 8.3
SaroE	19.8 ± 16.6	112.8 ± 12.9
KgalE	12.9 ± 24.1	33.2 ± 9.4
CDCA	33.2 ± 4.5^∗^	34.6 ± 8.6

### Microbial Transformation of CDCA and Plant Extracts

To simulate the passage of orally ingested compounds through the gastrointestinal system, the positive control CDCA and the prepared extracts were first suspended in SGF and incubated for 90 min to account for the physiological retention time in the stomach. After neutralization of this mixture with sodium hydroxide, either (i) deactivated fecal microbiota (i.e., non-transformed, labeled with superscript 0), or (ii) active fecal microbiota (i.e., transformed, superscript FS) from mice were added to facilitate the biotransformation of the constituents.

**FIGURE 3:**
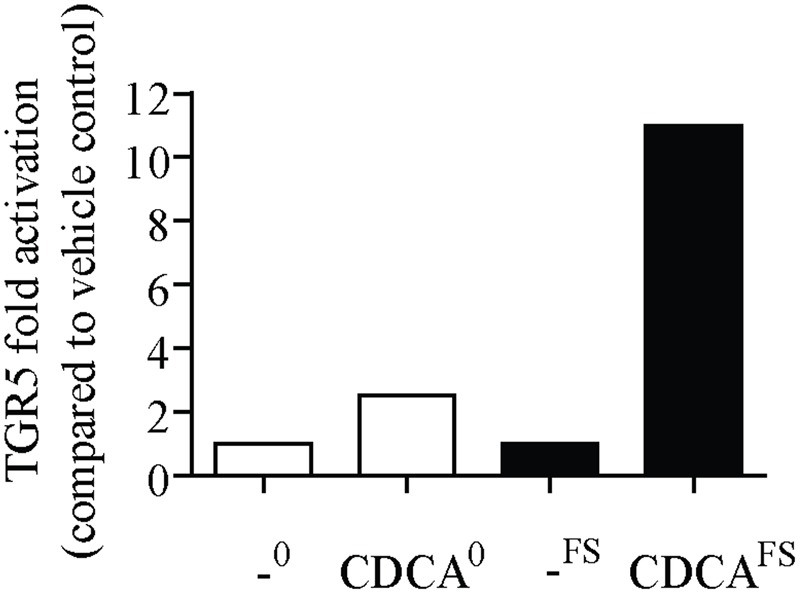
Microbial transformation of chenodeoxycholic acid (CDCA) to lithocholic acid (LCA) increases TGR5 activity. CDCA was incubated with (CDCA^FS^) or without fecal microbiota (CDCA^0^) from mice for 24 h. Corresponding vehicle controls are labeled “-^FS^” and “-^0^”, respectively. After centrifugation and sterile filtration, a TGR5 luciferase reporter gene assay was performed as described in the Section “Materials and Methods.” Cells were treated with 50 μM of CDCA^0^ and CDCA^FS^, respectively, for 18 h. The measured luciferase-derived luminescence was normalized to the obtained EGFP-derived fluorescence. Results are expressed as fold activation compared to the vehicle control. Each bar represents one experiment performed in octuplicates.

Incubation of the positive control CDCA with mouse fecal microbiota led to a strong biotransformation, thus proving the suitability of the experimental workflow. HPLC-DAD-CAD and LC-MS analyses showed that about half of the CDCA was consumed after 24 h while at least four different metabolites were produced (Supplementary Figure S1), presumably by 7-dehydroxylation, 3- or 7-dehydrogenation, and 3- or 7-epimerization as previously described (Gerard, 2013). After 24 h incubation, the most abundant metabolite was identified as LCA by comparison with a reference standard. LCA is the most potent endogenous agonist for TGR5 with CDCA being significantly less active (Kawamata et al., 2003). To evaluate our assay system, CDCA was incubated with active and deactivated fecal slurry for 24 h (CDCA^FS^ and CDCA^0^, respectively). In accordance with the observed transformation, CDCA^FS^ elicited a much higher response than CDCA^0^ in the TGR5 luciferase reporter gene assay (**Figure 3**).

Instrumental analysis of the transformed (KgalE^FS^, SaroE^FS^, and PdioE^FS^) and non-transformed extracts (KgalE^0^, SaroE^0^, and PdioE^0^) revealed that only PdioE and KgalE showed differences in the metabolite profile after transformation. In both cases, an increase in the abundance of compounds that were tentatively identified as free fatty acids and hydroxylated or otherwise oxidized fatty acids was detected, which can be explained by the action of lipolytic enzymes on remaining polar lipids in the extracts. For PdioE, it was additionally observed that a compound tentatively identified as ericifolin (Shamaladevi et al., 2013) by high-resolution mass spectrometry (HRMS) and fragment ion spectra was completely degraded after 24 h of incubation with mouse fecal microbiota. The comparison of KgalE^0^ and KgalE^FS^ showed a marked increase in the abundance of 4-methoxycinnamic acid, presumably by hydrolysis of larger phenylpropanoid structures, and higher concentrations of some not unambiguously identified small polar metabolites.

To elucidate a possible impact caused by incubation with fecal slurry, KgalE and PdioE were retested for their TGR5-activating potential, once incubated with active fecal slurry and once prepared with deactivated fecal slurry. However, other than in the case of CDCA, no significant difference in activity using the TGR5 reporter gene assay could be observed between KgalE^0^ and KgalE^FS^, and PdioE^0^ and PdioE^FS^, suggesting that the analytically observed biotransformation has no influence on the TGR5 activity. Therefore, we further focused on the identification of plant metabolites not targeted and modified by the fecal microbiota.

### Dereplication of the Main Constituents in the Active Extracts KgalE, SaroE, and PdioE

To identify the compounds responsible for TGR5 activity, all three active extracts were analyzed by HPLC and LC-MS. The accurate masses of the individual compounds were determined by HPLC-HRMS on an ESI-QqTOF system. Sum formulas were predicted based on mass accuracy (Δ*m/z* ≤ 10 ppm) and isotopic pattern matching. Finally, structural information was obtained by HPLC-multistage MS (MS^n^) on an ESI-ion trap system in DDA mode. Since all three plant species represent prominent spices and herbal drugs, they are well studied in terms of their chemical constituents. Thus, the main compounds could be tentatively identified as known metabolites of the investigated plant parts (see **Tables 3–5** and Supplementary Figures S2–S4 in the Supporting Information). The assignment of several of these structures was confirmed by comparison of the retention time and spectral data with reference standards.

**Table 3:** Proposed structure, retention time, HRMS data, and predicted sum formulas of the compounds tentatively identified in the extract of the dried flower buds of *Syzygium aromaticum* (SaroE).

#	Proposed structure	R_t_ (min)	[M + H]^+^	[M - H]^-^	Sum formula
S1	Gallic acid	2.4	171.0300	169.0146	C_7_H_6_O_5_
S2	Isobiflorin	5.5	355.1046	353.0889	C_16_H_18_O_9_
S3	Biflorin	5.9	355.1045	353.0889	C_16_H_18_O_9_
S4	Eugenol	16.1	165.0921	n.d.	C_10_H_12_O_2_
S5	Trihydroxy triterpene acid	17.5	489.3574	487.3440	C_30_H_48_O_5_
S6	Acetyleugenol	18.2	207.1029	n.d.	C_12_H_14_O_3_
S7	Maslinic acid	21.1	473.3647	471.3492	C_30_H_48_O_4_
S8	Coumaroylmaslinic acid	24.6	619.4007	617.3861	C_39_H_54_O_6_
S9	Oleanolic acid	26.6	457.3689	455.3541	C_30_H_48_O_3_
S10	Linoleic acid	28.5	n.d.	279.2340	C_18_H_32_O_2_
S11	Palmitic acid	31.0	n.d.	255.2337	C_16_H_32_O_2_
S12	Oleic acid	31.4	n.d.	281.2495	C_18_H_34_O_2_
S13	Stearic acid	34.3	n.d.	283.2650	C_18_H_36_O_2_

**Table 4:** Proposed structure, retention time, HRMS data, and predicted sum formulas of the compounds tentatively identified in the extract of the unripe fruits of *Pimenta dioica* (PdioE).

#	Proposed structure	R_t_ (min)	[M + H]^+^	[M - H]^-^	Sum formula
P1	Gallic acid	2.4	171.0299	169.0148	C_7_H_6_O_5_
P2	Hexosylated derivative of P4	4.8	361.1504	359.1359	C_16_H_24_O_9_
P3	Hexosylated derivative of P4	5.1	361.1502	359.1360	C_16_H_24_O_9_
P4	2′,3′-Dihydro-2′,3′-dihydroxyeugenol	5.1	n.d.^1^	197.0825	C_10_H_14_O_4_
P5	–	7.9	389.1821	387.1677	C_18_H_28_O_9_
P6	–	9.1	227.1296	225.1140	C_12_H_18_O_4_
P7	Ericifolin	14.3	495.1524	493.1373	C_23_H_26_O_12_
P8	Maslinic acid	27.5	473.3648	471.3500	C_30_H_48_O_4_
P9	Corosolic acid	27.8	473.3648	471.3502	C_30_H_48_O_4_
P10	Linolenic acid	31.9	279.2334	277.2188	C_18_H_30_O_2_
P11	Oleanolic acid	32.4	457.3689	455.3545	C_30_H_48_O_3_
P12	Ursolic acid	32.4	457.3689	455.3545	C_30_H_48_O_3_
P13	Linoleic acid	34.1	281.2491	279.2342	C_18_H_32_O_2_
P14	Palmitic acid	36.4	257.2493	255.2341	C_16_H_32_O_2_
P15	Oleic acid	36.7	n.d.	281.2499	C_18_H_34_O_2_
P16	Stearic acid	39.9	n.d.	283.2653	C_18_H_36_O_2_

*^1^The [M + Na]^+^ at *m/z* 221.0797 was detected instead.*

**Table 5:** Proposed structure, retention time, HRMS data and predicted sum formulas of the compounds tentatively identified in the extract of the rhizomes of *Kaempferia galanga* (KgalE).

#	Proposed structure	R_t_ (min)	[M + H]^+^	[M - H]^-^	Sum formula
K1	4-Methoxycinnamic acid	15.2	179.0716	177.0552	C_10_H_10_O_3_
K2	4-Hydroxycinnamic acid ethyl ester	17.8	n.d.	191.0711	C_11_H_12_O_3_
K3	3,4-Dimethoxycinnamic acid ethyl ester	21.4	237.1144	n.d.	C_13_H_16_O_4_
K4	4-Hydroxycinnamic acid propyl ester	23.1	207.1035	n.d.	C_12_H_14_O_3_
K5	4-Methoxycinnamic acid ethyl ester	24.5	207.1036	n.d.	C_12_H_14_O_3_
K6	–	27.0	231.1400	n.d.	C_15_H_18_O_2_
K7	–	27.3	n.d.	723.3802	C_34_H_60_O_16_
K8	Linoleoyl-glycero-3-phosphocholine	27.9	520.3429	n.d.^1^	C_26_H_51_NO_7_P
K9	Linoleic acid	39.9	281.2497	279.2331	C_18_H_32_O_2_
K10	Palmitic acid	42.6	n.d.	255.2331	C_16_H_32_O_2_
K11	Oleic acid	43.1	n.d.	281.2487	C_18_H_34_O_2_
K12	Stearic acid	46.6	n.d.	283.2645	C_18_H_36_O_2_

*^1^The [M + HCOO]^-^ at *m/z* 564.3304 was detected instead.*

All three extracts contained common fatty acids (e.g., palmitic, stearic, oleic, and linolenic acids) as well as cyclic oligomers of 𝜀-caprolactam—the latter being non-TGR5-influencing artifacts from the extract preparation.

**FIGURE 4:**
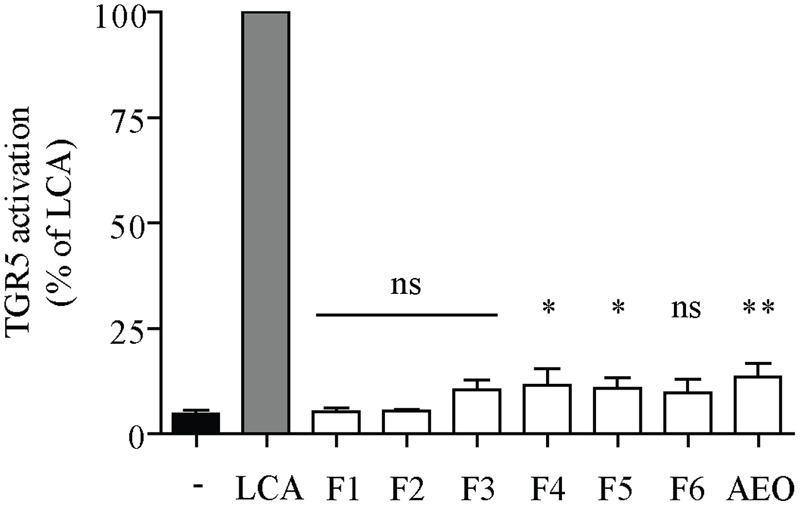
Fractionation of KgalE could not locate TGR5 activity in a specific fraction. HEK-293 cells were cotransfected with a TGR5 expression plasmid, a CRE luciferase reporter plasmid and an EGFP plasmid as internal control. Cells were treated with 10 μM lithocholic acid (LCA) as positive control or the 30 μg/mL of KgalE fractions (F1–F6) or essential oil (AEO) for 18 h. The measured luciferase-derived luminescence was normalized to the obtained EGFP-derived fluorescence. Results are expressed as % activation compared to the reference LCA. Each bar represents the mean ± SD of three independent experiments performed in quadruplicate and evaluated by unpaired Student’s *t*-test. ^∗∗^*p* < 0.01, ^∗^*p* < 0.05 compared with control (DMSO); ns, not significant versus control.

The HPLC-DAD-CAD and LC-MS analyses of KgalE indicated the presence of essential oil ingredients (Supplementary Figure S2) with the main component ethyl *p*-methoxycinnamate and the less abundant ethyl cinnamate (Wong et al., 1992; Raina and Abraham, 2016). Related derivatives, such as the corresponding propyl-esters and *p*-methoxycinnamic acid as well as fatty acid derivatives and minor unknown compounds were additionally detected (**Table 3**). Since none of these constituents have been described as TGR5 activators or are structurally similar to known TGR5 binders, KgalE was fractionated by RP-18 column chromatography to further assign the observed activity to a specific compound class or constituent. GC-MS analysis of the essential oil, which was obtained by steam distillation, revealed 92.2% *p*-methoxycinnamate and 1.3% ethyl cinnamate. The obtained six fractions as well as the steam distilled essential oil of *K. galanga* rhizomes were retested in the TGR5 luciferase reporter gene assay. Interestingly, the TGR5 activity was found to be distributed mainly between the essential oil and fractions 4 and 5 of KgalE (**Figure 4**). Thereby, the previously observed TGR5-activating potential of the extract could not be acuminated in one of these fractions. Moreover, the bioactive fractions negatively influenced cell viability, therefore we refrained from further separation and isolation of their constituents.

The extracts of *S. aromaticum* (SaroE) and *P. dioica* (PdioE) shared some similarities in their phytochemical profile (**Tables 3, 4**). Both samples contained some volatile and non-volatile phenolic compounds, such as isobiflorin, biflorin, eugenol, and acetyleugenol in SaroE, several dihydroxyeugenol derivatives and ericifolin in PdioE, and gallic acid in both. More importantly, TTAs were identified as major constituents of the extracts. The common monohydroxy TTAs betulinic, oleanolic, and ursolic acid were found to activate TGR5 with EC_50_ values in the range of 1–3 μM in previous studies (Sato et al., 2007; Genet et al., 2010). Indeed, comparison with reference compounds revealed oleanolic acid as the base peak in the CAD chromatogram of SaroE and both, oleanolic and ursolic acids as major constituents of PdioE. In addition, maslinic acid (in SaroE and PdioE) and corosolic acid (in PdioE), which are the 2α-hydroxy-derivatives of oleanolic acid and ursolic acid, respectively, were identified as prominent ingredients of these two extracts (**Figure 1**). Whether and how the additional hydroxy-group in 2α-position affects the activity on TGR5 has not been assessed so far.

### Quantification and TGR5 Activation of the Triterpene Acids

**FIGURE 5:**
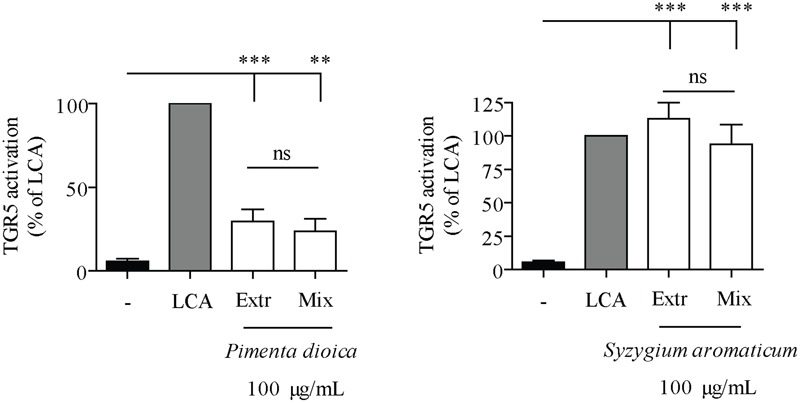
Pentacyclic triterpene acids in the extracts of *S. aromaticum* (SaroE) and *P. dioica* (PdioE) are the components activating TGR5. HEK-293 cells were cotransfected with a TGR5 expression plasmid, a CRE luciferase reporter plasmid and an EGFP plasmid as internal control. Cells were treated with 10 μM lithocholic acid (LCA) as positive control or 100 μg/mL of extracts (Extr) or reconstituted TTA mixtures (Mix) for 18 h. The measured luciferase-derived luminescence was normalized to the obtained EGFP-derived fluorescence. Results are expressed as % activation compared to the reference LCA. Each bar represents the mean ± SD of four independent experiments performed in quadruplicate and evaluated by unpaired Student’s *t*-test. ^∗∗∗^*p* < 0.001, ^∗∗^*p* < 0.01 compared with solvent vehicle control (DMSO); ns, not significant versus indicated group.

To verify the assumption that the measured TGR5 activity of SaroE and PdioE can be related to the identified pentacyclic TTAs, we developed a quantitative HPLC-CAD method with external standardization to determine their concentration in the tested extracts. Our method showed good linearity in the range of 2–100 μg/mL for each of the four compounds. SaroE was found to contain 6.56 ± 0.20% oleanolic acid and 2.70 ± 0.04% maslinic acid. In correlation with the lower activity, the concentration of the TTAs was less in PdioE, namely 0.290 ± 0.019% maslinic acid, 0.256 ± 0.012% ursolic acid, 0.198 ± 0.010% corosolic acid, and 0.148 ± 0.007% oleanolic acid. Based on these quantification results, reconstituted mixtures of the identified TTAs were prepared in the same concentration and composition as in the final test concentration (100 μg/mL) of SaroE, i.e., 14.4 μM oleanolic acid and 5.72 μM maslinic acid, and PdioE, i.e., 0.32 μM oleanolic acid, 0.61 μM maslinic acid, 0.42 μM corosolic acid, and 0.56 μM ursolic acid. These reconstituted mixtures, together with the individual pure compounds, were tested in the TGR5 luciferase reporter gene assay. In both cases, the reconstituted mixtures of TTAs were equally potent compared to the respective extracts (**Figure 5**). The EC_50_ and *E*_max_ values of the extracts, the reconstituted mixtures and the single compounds are listed in **Table 6**.

**Table 6:** EC_50_ and *E*_max_ values of indicated extracts, reconstituted mixtures of TTAs and pure compounds.

	EC_50_ (μg/mL) or (μM)^∗^	*E*_max_ (% LCA)
SaroE	19.8 ± 16.6	112.8 ± 12.9
PdioE	60.2 ± 13.7	29.5 ± 8.3
TTA (SaroE)	40.7 ± 40.1	93.7 ± 16.2
TTA (PdioE)	80.7 ± 36.6	23.6 ± 9.3
Oleanolic acid	2.2 ± 1.6*	62.6 ± 14.9
Maslinic acid	3.7 ± 0.7*	29.2 ± 5.0
Corosolic acid	0.5 ± 1.0*	9.8 ± 3.3
Ursolic acid	1.1 ± 0.2*	19.6 ± 14.2

## Discussion

Multi-component herbal preparations are widely used in traditional medicines. Deciphering whether the activity of such a complex mixture can be attributed to individual constituents is a widespread research interest.

In the present study, we investigated 19 plant extracts from a traditional Tibetan formula used against several symptoms related to the metabolic syndrome for their TGR5-activating potential. Out of these extracts, we identified three which were able to significantly upregulate TGR5 activity in a cell-based luciferase reporter gene assay. Interestingly, the active extracts, SaroE, PdioE, and KgalE, are derived from the three commonly used spices, namely clove (flower buds of *S. aromaticum*), allspice (*P. dioica*), and aromatic ginger (rhizome of *K. galanga*), respectively.

Among them, SaroE activated TGR5 at 100 μg/mL even stronger than the positive control LCA at 10 μM. *S. aromaticum* extracts and its triterpene constituents have been reported to elicit many beneficial effects, for instance antihyperglycemic, antihyperlipidemic, and antioxidant activity as well as a reduced formation of cholesterol micelles *in vitro* (Sompong et al., 2016). Furthermore, the *S. aromaticum*-derived triterpenes oleanolic acid and maslinic acid have been shown to improve glucose homeostasis and suppress postprandial hyperglycemia in diabetic rats via inhibition of carbohydrate hydrolysis and reduction of glucose transporters in the gastrointestinal tract (Khathi et al., 2013). The other two plant extracts activating TGR5 in our assay system are PdioE and KgalE. Extracts from *P. dioica* have been reported to have hypotensive activity (Suarez et al., 1997). Moreover, traditional use for *P. dioica* suggests a beneficial effect for gastrointestinal and metabolic disorders as it is used for dyspepsia and diabetes, to relieve indigestion and as purgative in different folk medicines (Zhang and Lokeshwar, 2012). *K. galanga* extracts have been shown to act anti-inflammatory (Jagadish et al., 2016) and its constituent ethyl cinnamate is reported to have vasorelaxant activity (Othman et al., 2006). Activation of TGR5 could play a role in several of these reported activities.

In the last years, the role of the gut microbiome in the metabolism and transformation of dietary compounds and orally administered pharmaceuticals gained more and more attention (Marin et al., 2015; Li H. et al., 2016; Sonnenburg and Backhed, 2016; Zhang and Davies, 2016). Naturally occurring gut metabolites of dietary sources or herbal medicinal preparations may be the active principles of many ethnopharmacologically used preparations. Many traditional herbal remedies are used orally: After ingestion, ingredients are released, occasionally metabolized, and then absorbed via the gut. Once in the blood stream, constituents can enter other tissues to interact with respective signal transduction pathways. Apart from this view, oral medicines may also be able to interact with receptors present in gastrointestinal cells thereby eliciting systemic effects without entering the blood stream (Reimann et al., 2012). This also renders the effect of pharmaceuticals independent on bioavailability. As orally administered herbal preparations are able to reach their highest concentration locally in the gut, membrane receptors like TGR5 are likely targets. Furthermore, constituents can be transformed by the microbiome present in the gut yielding active metabolites. An example for such an action is the transformation of CDCA to LCA. CDCA activates FXR in the ileum but after bacterial transformation to LCA it is a far better activator for TGR5 localized mainly in intestinal L-cells in the large intestine (Hirano et al., 1981).

To study a possible microbial transformation of extract constituents, we incubated the three TGR5-activating plant extracts SaroE, PdioE, and KgalE with mouse fecal microbiota. The transformation of CDCA to LCA by incubation with fecal samples from mice was used to validate our assay system. Instrumental analysis revealed that only PdioE and KgalE but not SaroE showed a changed metabolite profile after transformation. Retesting of the transformed PdioE and KgalE extracts, however, did not alter the TGR5 activation profile. We therefore focused on the identification of non-transformed constituents in SaroE, KgalE, and PdioE.

Dereplication, quantification, and retesting of the extracts’ constituents clearly deciphered TTAs as the only TGR5 effectors in the multicomponent mixtures of SaroE and PdioE. Analysis and retesting of KgalE fractions, however, could not trace back the activity exclusively to a specific compound or compound class suggesting an interaction of several constituents, such as volatile and non-volatile phenylpropanoids, that contribute to the observed effect.

Whereas oleanolic acid and ursolic acid have already been reported to activate TGR5 with EC_50_ values between 1 and 3 μM (Sato et al., 2007; Genet et al., 2010), we could further ascribe the TGR5-upregulating activity to the TTAs corosolic acid and maslinic acid. Many studies have been performed investigating corosolic acid (Miura et al., 2006; Yamaguchi et al., 2006; Yamada et al., 2007; Shi et al., 2008; Chen et al., 2012; Li et al., 2014; Li X.Q. et al., 2016; Yang et al., 2016) and maslinic acid (Wen et al., 2005, 2008; Liu et al., 2007, 2014; Guan et al., 2011; Khathi et al., 2013; Mkhwanazi et al., 2014; Hung et al., 2015) with regard to their beneficial impact on diabetes and the metabolic syndrome. In respect of the structural similarities to oleanolic acid and ursolic acid, maslinic acid and corosolic acid are likely to activate TGR5 in a similar manner.

Structure–activity studies of pentacyclic TTAs identified the C3-hydroxyl and the C28-carboxylate group as being of major importance for TGR5 activation. Oleanolic acid, ursolic acid, corosolic acid, and maslinic acid all contain these two features (**Figure 1**), thereby suggesting the same binding mode. It has been predicted that triterpenes bind TGR5 via three binding pockets. Recognition of the C3-hydroxyl group is accomplished via a narrow H-bonding site. The pentacyclic skeleton is harbored in a hydrophobic pocket and a small polar site binds the free carboxylic acid (Genet et al., 2010; Castellano et al., 2013).

The fact that the only TGR5 agonist that has been studied in humans so far, SB-756050, has thus-far failed to be launched, underlines the need for TGR5 activators with a favorable benefit:risk ratio. SB-756050 elicited variable pharmacodynamic effects at different doses on circulating GLP-1 and PYY in humans. This was in contrast to results obtained in animals and questioned the general translatability to humans (Hodge et al., 2013). Moreover, intestinally targeted TGR5 agonists are at the moment discussed to be better suited for therapeutic use (Cao et al., 2016), making compounds with low oral bioavailability, like oleanolic acid, interesting (Jiang et al., 2016). In this respect, herbal remedies and food supplements may offer highly interesting sources for adequate concentrations of the hereby identified TTAs that are able to locally target and upregulate TGR5 in intestinal L-cells of the large intestine.

## Conclusion

This is the first study that reports the TGR5-upregulating effect of the herbal remedies and condiments aromatic ginger, allspice, and clove. This may explain their therapeutic potential for the treatment of metabolic diseases. Moreover, the TGR5 activation of SaroE and PdioE could be pinpointed solely to the TTAs oleanolic acid and ursolic acid and the novel TGR5 agonists maslinic acid and corosolic acid.

## Author Contributions

JR, VD, DB, AL, and MZ conceived and designed the experiments. AL, CH, NF, and FP performed the experiments. AL, MZ, and UG analyzed the data. AL wrote the manuscript. MZ, UG, VD, and JR critically revised the manuscript. All authors have contributed to the final version and approved the final manuscript.

## Conflict of Interest Statement

The authors declare that the research was conducted in the absence of any commercial or financial relationships that could be construed as a potential conflict of interest.
