# Supplementary material for: Allspice and Clove As Source of Triterpene Acids Activating the G Protein-Coupled Bile Acid Receptor TGR5
Source: Front Pharmacol. 2017 Jul 17;8:468. doi: 10.3389/fphar.2017.00468 (PMC5511840; doi:10.3389/fphar.2017.00468)
Supplement: Supplementary file 1 [file Data_Sheet_1.DOCX]

**Supplemental Figures:**

**Figure Legends:**

**Figure S1: Biotransformation of chenodeoxycholic acid (CDCA) by mouse fecal microbiota.**

HPLC-CAD chromatograms of CDCA after incubation with either sterile, inactive (A) or fresh, active (B) mouse fecal slurry. CDCA and lithocholic acid (LCA) were identified by comparison with reference compounds while the structures of the other three metabolites were tentatively assigned based on their masses and known biotransformation pathways.

**Figure S2: Dereplication of the main constituents in the extract of the dried flower buds of *Syzygium aromaticum* (SaroE).**

HPLC-CAD (A) and HPLC-DAD (λ=200 nm) (B) chromatograms of SaroE. The compounds S1-S13 were tentatively identified by ESI-Qq-TOF-MS and ESI-ion trap-MS and the corresponding data are shown in Table 3. Peaks labeled with an asterisk (*) represent impurities (e.g. the Nylon-derived cyclic oligomers of ε-caprolactam eluting after 9-13 min).

**Figure S3: Dereplication of the main constituents in the extract of the unripe fruits of *Pimenta dioica* (PdioE).**

HPLC-CAD (A) and HPLC-DAD (λ=200 nm) (B) chromatograms of PdioE. The compounds P1-P16 were tentatively identified by ESI-Qq-TOF-MS and ESI-ion trap-MS and the corresponding data are shown in Table 4. Peaks labeled with an asterisk (*) represent impurities (e.g. the Nylon-derived cyclic oligomers of ε-caprolactam eluting after 11-16 min).

**Figure S4: Dereplication of the main constituents in the extract of the rhizomes of *Kaempferia galanga* (KgalE).**

HPLC-CAD (A) and HPLC-DAD (λ=200 nm) (B) chromatograms of KgalE. The compounds K1-K12 were tentatively identified by ESI-Qq-TOF-MS and ESI-ion trap-MS and the corresponding data are shown in Table 5. Peaks labeled with an asterisk (*) represent impurities (e.g. the Nylon-derived cyclic oligomers of ε-caprolactam eluting after 9-13 min).

**Figure S1:**

**Figure S2:**

**Figure S3:**

**Figure S4:**
